# From Ebola to H5N1: Strengthening the U.S. Special Pathogen Response System

**DOI:** 10.3390/epidemiologia7030079

**Published:** 2026-06-04

**Authors:** Anthony Joseph Lo Piccolo, Erin McGuire, Radu Postelnicu, Kathryn Jano, Ryan Leone, Eliana Jacobson, Angela Vasa, Michelle Schwedhelm, Vikramjit Mukherjee

**Affiliations:** 1 Department of Medicine, New York University Grossman School of Medicine, New York, NY 10016, USA; 2 Special Pathogens Program, NYC Health + Hospitals/Bellevue Hospital, New York, NY 10016, USA; 3 New York Medical College School of Medicine, Touro University, Valhalla, NY 10595, USA; 4 Vagelos College of Physicians and Surgeons, Columbia University, New York, NY 10032, USA; rml2207@cumc.columbia.edu; 5 National Institute for Defense Health Cooperation, Uniformed Services University, Bethesda, MD 20814, USA; 6 Emergency Preparedness and Special Pathogen Programs, Nebraska Medical Center: Global Center for Health Security Scholar, University of Nebraska Medical Center, Omaha, NE 68198, USA; avasa@nebraskamed.com; 7 Emergency Management and Clinical Operations, University of Nebraska Medical Center, Omaha, NE 68198, USA

**Keywords:** special pathogens, RESPTC, NETEC, H5N1, HCID, pandemic preparedness, frontline hospitals, high-consequence infectious diseases, NSPS, Ebola virus

## Abstract

The National Special Pathogens System (NSPS) stratifies U.S. healthcare facilities by their readiness level to care for patients with high-consequence infectious diseases (HCIDs). While NSPS Level 1 and 2 facilities possess advanced biocontainment capabilities to care for patients for the duration of their illness, most U.S. hospitals fall under a NSPS Level 3 or 4 designation, with limited resources to manage patients with a suspected or confirmed HCID. However, emerging zoonotic threats like H5N1 underscore the need to bolster HCID preparedness across all NSPS Levels. Beginning in March 2024, the U.S. H5N1 outbreak has primarily impacted wild bird flocks, poultry, and cattle, along with some human infections. The continuation of this outbreak in wild and domesticated animals increases the likelihood of further human spillover and eventual viral evolution in human hosts. At the frontlines, rural farming communities are likely to be most affected, with potential outbreaks exacerbated by a lack of accessible NSPS Level 1, 2, or 3 facilities in these regions. Thus, strengthening the HCID preparedness of local NSPS Level 4 facilities is critical to preventing transmission, minimizing societal disruption, protecting communities and the healthcare workforce, along with ensuring an equitable, coordinated response to future emerging infectious disease threats. This manuscript explores the financial, societal and health system impacts of HCID outbreaks to delineate the necessity of strengthening the preparedness of NSPS Level 4 facilities.

## 1. Introduction

**Figure 1:**
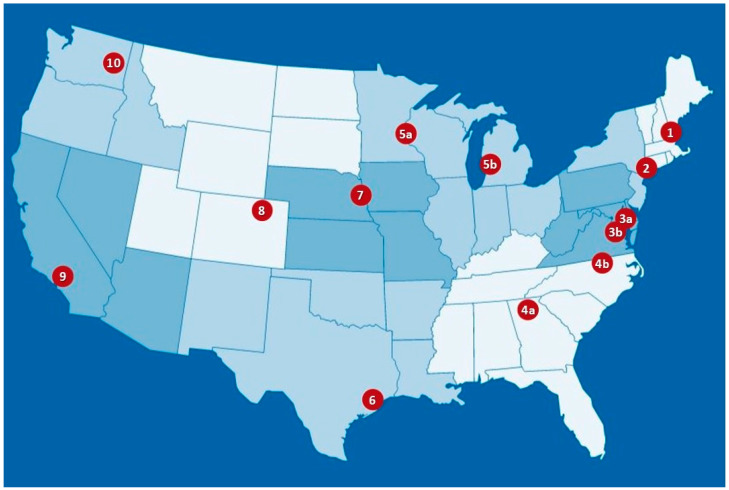
United States map of the 13 Regional Emerging Special Pathogen Treatment Centers (RESPTCs). This map displays the location of the 13 Regional Emerging Special Pathogen Treatment Centers (RESPTCs) and the regions they serve. 1: Massachusetts General Hospital. 2: NYC Health + Hospitals/Bellevue. 3a: Johns Hopkins Hospital. 3b: MedStar Washington Hospital Center/Children’s National. 4a: Emory University/Children’s Healthcare of Atlanta. 4b: University of North Carolina at Chapel Hill. 5a: University of Minnesota Medical Center. 5b: Corewell Health System. 6: University of Texas Medical Branch. 7: University of Nebraska Medical Center/Nebraska Medicine. 8: Denver Health and Hospital Authority. 9: Cedars Sinai Medical Center. 10: Providence Sacred Heart Medical Center and Children’s Hospital.

A network of regional special pathogen treatment centers was first realized after the 2014–2016 Ebola virus outbreak, during which U.S. responders treated 11 patients for Ebola virus disease [1]. Originally coined the Regional Ebola Treatment Network (RETN), the Ebola treatment centers were established by the U.S. Health and Human Services (HHS) Administration for Strategic Preparedness and Response (ASPR) in 2015 and consisted of 10 centers [2]. In 2019, the RETN was renamed the Regional Emerging Special Pathogen Treatment Centers (RESPTCs), generalizing to all high-consequence infectious diseases (HCIDs) [3]. As of 2025, there are 13 ASPR-designated and funded RESPTCs (Figure 1) capable of receiving and caring for patients with suspected or confirmed HCID infections [4]. All RESPTCs are equipped with necessary supplies and have optimized structural accommodations, including designated units with negative-pressure patient care rooms, biocontainment-compatible clinical devices, biocontainment laboratories, trained staff, and personal protective equipment (PPE) [5]. Additional preparedness measures include quarterly staff training on properly donning and doffing PPE aligned to pathogen transmission routes, implementation of infection prevention and control strategies, and developing best practices to care for patients with HCIDs.

Although the Ebola virus outbreak was successfully contained and managed by a small group of specialized hospitals, which later formed the RESPTC network, the COVID-19 pandemic exposed the limited capacity of the national infectious disease outbreak response system [6]. While the RESPTCs are adequately prepared to treat patients with HCIDs and contain outbreaks, a highly contagious and/or infectious pathogen can quickly overwhelm the network, requiring a distinct national management approach. Such a pathogen requires a coordinated and nimble response that stretches across the healthcare continuum, inspiring the development of the National Special Pathogens System (NSPS) [3]. Much like the Trauma Center level designation, the NSPS stratifies healthcare facilities into four levels by their ability to treat a patient with a suspected or confirmed HCID [7]. The National Emerging Special Pathogens Training and Education Center (NETEC) has been congressionally appointed to be NSPS’s coordinating body and has defined the expected minimum capabilities for each level during an HCID response, designed to standardize care delivery expectations [8,9].

NSPS Level 1 facilities are RESPTCs and represent the most advanced level of HCID care in the country. They are prepared to deliver specialized care to two or more patients with a viral hemorrhagic fever (VHF; i.e., viruses in the Arenaviridae, Filoviridae, Bunyaviridae, and Flaviviridae families such as Ebola virus, Marburg virus, and Lassa virus, among others), and 10 or more patients with an airborne HCID. RESPTCs also serve their Health and Human Services (HHS) region during both preparedness and response periods by disseminating information on current HCID events, providing technical assistance for NSPS partners, delivering education and training, and promoting NETEC program assessment tools and best practices to NSPS hospitals, healthcare coalitions, public health departments, and EMS agencies. NSPS Level 2 facilities can deliver specialized care and support to 1–2 VHF patients, and four or more patients with an airborne pathogen throughout their illness course. NSPS Level 3 facilities consist of assessment centers that have the capacity to stabilize a patient with supportive care, conduct limited clinical laboratory testing and coordinate access to HCID diagnostic testing. Lastly, all remaining healthcare facilities are considered NSPS Level 4 and, at a minimum, are expected to safely identify patients that may have an HCID, isolate them, and inform internal and external partners (including local health authorities) of suspected HCID infections.

In July 2024, The Joint Commission– an independent not-for-profit organization that accredits and certifies healthcare organizations in the United States—released new infection prevention and control standards and elements of performance for HCIDs within their infection control chapter. The inclusion of these elements of performance elevates the expectation for all healthcare facilities accredited by The Joint Commission to be prepared to perform these functions. NSPS Level 3 and 4 facilities are not expected to provide definitive care to a patient with a confirmed HCID infection. Thus, these facilities are generally charged with identifying a potential HCID infection, providing stabilizing care, collecting specimens for diagnostics and supporting clinical testing (Level 3 facilities), and safely transferring the patient to a higher-tiered NSPS facility as appropriate. See Table 1 for further delineation between the NSPS Level designations [10].

Funding for special pathogen preparedness varies widely based on NSPS Level and location. NSPS Level 1 facilities are funded by ASPR to support both in-house special pathogen preparedness efforts as well as outreach and preparedness for their respective regions, much like Level 1 Trauma Centers. Level 2 facilities may receive funding from ASPR through the Hospital Preparedness Program (HPP), which is awarded to state health departments to maintain their preparedness levels. Some Level 2 facilities do not receive HPP funding; however, through internal healthcare facility support, they maintain their NSPS Level 2 status. In 2025, NETEC received federal funding to award up to 75 new NSPS Level 2 facilities across the country [11]. While this will help strengthen the NSPS, it is a one-time infusion of funding for the Level 2 facilities. As has been seen in the past, without sustained funding, many hospitals struggle to maintain the essential functions to serve as a Level 2 facility long-term. NSPS Level 3 Assessment Centers may receive HPP funding via their state health department, although this is not consistent across the U.S. and often becomes a barrier to reaching the respective minimum capabilities. Level 4 hospitals, which encompass all other healthcare facilities, do not receive special pathogen preparedness funding.

Most Level 1 and Level 2 NSPS facilities are in urban settings, leading to a focus on pandemic preparedness tabletops and drills in these areas; however, compounding factors in rural communities may place them at the frontlines of a special pathogen outbreak. Primarily, most American agricultural production occurs on large, specialized farms in rural areas, where one quarter of the population lives [12]. These agricultural sites create animal–human interfaces for infectious pathogens to transmit and evolve in a human host [13], with the spread of SARS-CoV-2 in rural Missouri serving as a case study [14]. Rural communities might also rely on swap meets, where live farm animals are traded and may further spread disease between regions [15]. Secondly, rural areas are historically medically underserved. With a lower density of healthcare providers and distant healthcare facilities [16], rural patients travel twice the distance to hospital centers compared to those from urban areas, on average [17]. Because of this, patients with a suspected or confirmed HCID infection often present later in their illness course to a healthcare facility, allowing for additional opportunities for extensive community-based transmission [18]. The expansion of the NSPS can help address these disparities and improve equitable access to HCID care for all Americans. The ability to locally care for patients with suspected or confirmed HCID infections will prove critical to achieve zero preventable deaths and minimize further transmission.

Transmission of the highly pathogenic avian influenza A (H5N1 virus) through contact between and amongst birds, cows, and humans, which has escalated across the U.S. since March 2024, clearly demonstrates the growing burden carried by rural communities in outbreak settings. As Americans increasingly move to rural areas and encroach upon animal habitats [19], expanding rural communities may be the canary in the coal mine for novel emerging diseases. Here, we examine how emerging HCIDs impact the healthcare system and the communities they serve, as well as provide insight into how NSPS Level 4 facilities can strengthen their HCID preparedness.

## 2. Costs of Patient Care for HCIDs

Treating patients with a suspected or confirmed HCID infection is costly. Without a tailored response, the costs of identification, treatment, and containment can increase exponentially. During the 2014–2016 Ebola virus outbreak, “Emory, which treated four patients, spent close to $1 million in direct costs to care for a single high-intensity patient,” said Gartland, then vice president of operations for Emory University Hospital [20]. The Nebraska Medical Center, which cared for three patients with a confirmed Ebola virus infection, said its direct costs from its first two patients surpassed $1 million [21]. As part of their infection control protocol, 10 hospital beds were removed from service. The vacant beds led to an “opportunity cost” of another $148,000 at the Nebraska Medical Center, according to hospital officials [21]. While costs were high, the federal government reimbursed the hospitals that cared for repatriated Americans during this outbreak. Nevertheless, repositioning healthcare staff to care for high acuity and contagious patients disrupts the typical and already overwhelmed workflow of U.S. hospitals.

In a similar example from Trauma Centers, the arrival of critically injured patients to an emergency department (ED) may disrupt standard services, as these admissions require healthcare teams to mount a rapid response with specialized staff to perform assessments and diagnostics, while initiating stabilizing care. Hospitals that are designated as Level 3–5 Trauma Centers, located in rural areas, or experiencing staffing constraints, are especially vulnerable. Similarly, when a patient with a suspected or confirmed HCID infection presents to an ED, standard procedures can be dramatically impacted as the patient is isolated, trained staff are identified and assigned, and the appropriate care interventions are initiated. NSPS Level 4 centers without established or regularly trained care teams, HCID infection control procedures, and/or proper supplies and infrastructure are at higher risk of experiencing impactful disruptions when HCID cases present to their facilities. The provision of safe and effective care for patients with a HCID is resource-intensive, requiring utilization of a designated space that is separated from other patient care spaces, staff who have completed training in the utilization of PPE and infection control practices, and supplies that may not be routinely used. NSPS Level 4 hospitals may not have established HCID protocols or healthcare teams trained in HCID care, in addition to inadequate resources to manage an outbreak response. The provision of care for patients suspected or confirmed to have a HCID in a resource-limited setting can disrupt ED workflows and negatively impact the ability of the team to provide timely care to other ED patients. Thus, to properly care for an HCID patient and the larger community, hospital resources must be effectively and equitably reallocated. This diversion of resources for a high-containment pathogen activation can be delineated by the four S’s: “Space” to safely isolate a patient, don and doff PPE, and temporarily hold Category A waste; “Staff” such as a trained multidisciplinary team of nurses, physicians, specialists, laboratory technicians, and other infection control personnel; “Stuff” including PPE, and laboratory equipment; and “System” referencing the procedural changes that must take place for decontamination, isolation, transportation, waste management, and other operational tasks [22].

As evidenced by the COVID-19 pandemic, novel emerging pathogen outbreaks continue to significantly threaten global health. As of 27 March 2026, there have been over 1.2 million deaths, and through April 2021, an estimated 3.5 million hospitalizations were attributed to SARS-CoV-2 across the United States [23,24]. This strain on healthcare systems carries a heavy financial burden. According to the American Medical Association, the U.S. National Health Expenditures (NHE) reached $4.1 trillion, an increase of 9.7% from 2019 [25]. Although SARS-CoV-2 was highly infectious, capable of causing severe disease with high mortality rates, and was initially considered an HCID before vaccines were developed and viral transmission dynamics and mortality were better understood, other pathogens have the potential to be as or more transmissible with an even higher mortality. Given the high direct and indirect costs of care for a single HCID patient, the expenses the healthcare system could incur in response to an uncontrolled HCID outbreak could easily surpass the $4.1 trillion level of expenditures seen in 2020.

Additionally, pandemic-induced healthcare worker burnout has had lasting impacts on the healthcare workforce, causing healthcare facilities to rely on contract labor and travel nurses to resolve major physician and registered nurse staffing challenges. While travel nurses can fill in staffing gaps to maintain patient-to-nurse ratios, they come at a premium cost. The Center for Economic and Policy Research found that travel nurses were making 103.3% more than their staff nurse counterparts in 2022 [26]. With limited nursing staff available, the price for temporary services soared. This effect can be exacerbated further by an HCID outbreak since these patients historically require higher nurse-to-patient ratios due to the advanced infection prevention practices, use of transmission-based PPE, and the increased risk to staff given the high morbidity of the pathogen(s).

**Figure 2:**
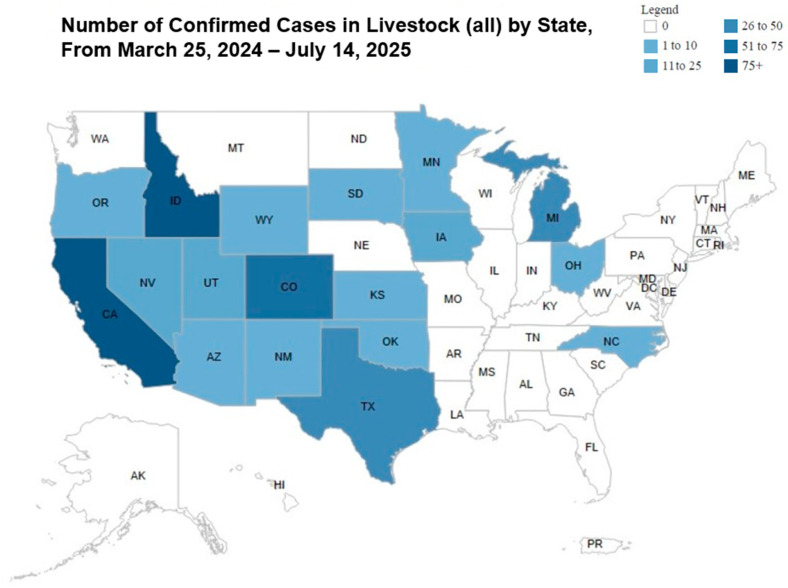
Confirmed cases of H5N1 in livestock from 25 March 2024 to 14 July 2025. The increasing number of U.S. livestock infected with H5N1 raises the risk of spillover into the human population.

Most recently, the U.S. H5N1 outbreak (Figure 2) beginning in March 2024 serves as an example of a One Health approach, which may be an effective framework to mitigate the exorbitant costs associated with HCID outbreaks [27]. One Health refers to an integrative and collaborative approach between healthcare, veterinary medicine, agriculture, and environmental sciences to tackle health risks and hazards, including emerging infectious diseases [28]. In May 2024, the USDA began providing up to $28,000 per premises with herds affected by H5N1 to prevent further spread, including $2000 per month for farmworker PPE, up to $1500 to develop and support biosecurity plans, up to $2000 per month to inactivate the virus in all waste milk via heat treatment, up to $10,000 for reimbursement of veterinary costs incurred for H5N1 treatment and sample collection, and $100 per month to offset shipping costs of influenza A testing [27]. HHS followed suit, investing a total of $101 million to mitigate H5N1 [27]. On 26 February 2025, U.S. Secretary of Agriculture Brooke Rollins announced a $1 billion comprehensive strategy to curb highly pathogenic avian influenza (HPAI) [29]. These tools support affected farms by increasing H5N1 monitoring and reducing routes of transmission. Addressing pathogens while they are transmitting in animal reservoirs moderates the risk of human spillover events [30].

## 3. Societal Impacts of an Outbreak

The psychosocial toll of outbreaks has been shown to detrimentally impact several facets of society, including the private business sector, education, and mental and physical health. In March 2020, the U.S. Congress passed the $2.2 trillion Coronavirus Aid Relief and Economic Security (CARES) Act to keep businesses, including hospitals, afloat during the COVID-19 lockdowns [31]. Additional legislation was passed through the next two years aimed at stimulating the economic recovery. Despite these policy efforts, employees and businesses still suffered during the pandemic, with full-time worker unemployment peaking at 14.8% in April 2020 and temporary or permanent closures of over 700,000 business establishments in the U.S. alone [32,33]. Schools and educational institutions also closed due to the COVID-19 pandemic, impacting roughly 1.6 billion students and almost 94% of the global student population [34]. Globally, while pediatric emergency room visits decreased, school closures have been associated with the lack of access to school-based healthcare, nutrition systems, and support in remote learning, as well as an increase in anxiety and loneliness and childhood obesity prevalence [35]. In the U.S., the federal government implemented mandatory closures of social safety nets, such as schools and implemented mass social-distancing measures [36]. The National School Lunch Program, which is one of the largest national food and nutrition assistance programs, was disrupted by school closures for the roughly 30 million children who depend on them [37,38].

Additionally, roughly 50% of the world’s population was initially instructed to avoid public spaces in the early stages of the pandemic [39]. The pandemic led to social isolation and loneliness for over 50% of Americans, particularly among young adults and the elderly [40]. This caused significant, prolonged distress, depression, and anxiety across the country [41]. Furthermore, the widespread use of masks during the COVID-19 pandemic reduced the spread of infection, but inadvertently changed societal norms around mask-wearing and potentially impacted psychosocial development in children, based on mixed results [42]. Mask-wearing and other public health measures, such as vaccination campaigns, also resulted in an insidious backlash against public health and a decrease in trust for healthcare providers; for example, vaccinations among those ages 2–18 dropped precipitously [43], likely in part due to logistical limitations while providers cared for COVID-19 patients. However, rates of routine vaccinations have remained lower than pre-pandemic levels, nonmedical school vaccination exemption rates have increased, and vaccine coverage for rural and poor children in particular has dropped 5% since 2020 [44,45]. A recent study showed that trust in physicians and hospitals dropped from 71.5% to 40.1% between April 2020 and January 2024, with lower COVID-19 vaccination rates among those showing lower levels of trust [46].

Beyond individuals, local governments have also raised backlash against public health efforts, as evidenced by the many state laws passed to restrict government acts during health emergencies, with four states prohibiting vaccine or proof of vaccination requirements and five prohibiting mask mandates [47], alongside a recent controversial ban on public mask-wearing that was passed in Nassau County, New York [48]. Reminiscent of the toilet paper shortage of the COVID-19 pandemic, the 2025 egg scarcity causing inflated egg prices, and the temporary closure of all NYC, Westchester, Suffolk, and Nassau counties live bird markets due to the H5N1 outbreak have demonstrated the continued societal impacts of HCIDs [49,50,51]. This real-world evidence suggests that future pandemics may also lead to massive disruptions in society across both urban and rural areas. The costs of being unprepared for future pandemics come in more forms than money alone can show.

## 4. Strengthening the National Special Pathogens System (NSPS)

During an outbreak, it is integral that all healthcare facilities are prepared to identify potential cases, quickly isolate applicable patients, and inform their local health department. The recent COVID-19 pandemic has highlighted the need for a standardized approach to hospital preparedness for HCIDs, especially in NSPS Level 4 facilities. Previously, there was little established guidance and no mandatory requirements for how these healthcare facilities should prepare for HCID patients. This has led The Joint Commission to develop new requirements, effective as of 1 July 2024 [52], aimed at enhancing hospitals’ preparedness for HCIDs.

At a minimum, The Joint Commission now requires all hospitals to develop HCID-specific protocols with a focus on Identify, Isolate, and Inform (III) guidelines. These standardized protocols will assist healthcare workers with initial screening and management of patients suspected of having a HCID. Identification protocols involve screening at all points of entry for respiratory symptoms, fever, rash, and travel history to identify potential patients with a HCID. It is important to consider that patients with a HCID may present at any hospital unit, or in the urgent care or ambulatory care setting. Training staff throughout the hospital with a basic understanding of HCID identification procedures will allow for earlier isolation of patients and prevention of unnecessary healthcare worker or patient exposure. Isolation protocols must consider transmission-based precautions and ensure hospital infrastructure can support those precautions. Protocols should clearly outline how to alert and inform key hospital staff, such as infection control or the hospital epidemiologist, and public health authorities of a patient with a possible HCID.

Additionally, The Joint Commission requirements call for infection control and waste management protocols. Infection control measures include defining the hospital’s HCID PPE ensemble, avoidance of aerosol-generating procedures, hand hygiene compliance and healthcare worker monitoring for potential exposures. These measures should be clearly protocolized to mitigate the risk of exposure to hospital staff, patients, and visitors. Waste management protocols should detail plans for managing Category A waste, and cleaning and disinfecting patient care spaces and equipment. Some healthcare facilities may have access to an on-site autoclave to inactivate their Category A waste. When on-site inactivation is not possible, waste must be properly packaged and transported by a vendor with the ability to apply for a special permit to an approved facility for inactivation or final disposition [53]. NETEC maintains The Joint Commission infection control standards resources page on its website to provide organizations with guidance on how to implement these new requirements [54].

The second element in the new Joint Commission requirements is the development of training and education for staff. While The Joint Commission does not define the cadence of training, training and education programs can be modeled after those existing at RESPTCs and developed by NETEC and then scaled to meet the individual needs of each organization [8]. RESPTCs provide quarterly training to the core HCID patient care team of ICU nurses and physicians, while lab staff, site managers, environmental services, and emergency department nurses and physicians are trained biannually. Training sessions concentrate on PPE donning and doffing procedures and often include practicing new or updated protocols to promote staff familiarity. To test and validate the HCID protocols and the effectiveness of the training program, quarterly drills are performed in close collaboration with the hospital’s emergency management team. The development of successful training programs in NSPS Level 4 facilities will provide an educated and well-informed staff, ultimately reducing potential exposures and thus transmission.

There are additional strategies beyond The Joint Commission requirements that NSPS Level 4 healthcare facilities can implement to advance and strengthen their HCID preparedness. First, patient transport workflows could be developed in adherence to infection control precautions. Once local public health authorities are notified, they will involve the local and state health departments and the region’s RESPTC to help guide the facility through the next steps to deliver safe patient care. Depending on the facility’s NSPS Level, it may be advised to transfer the suspected patient to a higher level of care. In preparation for this recommendation, hospitals should develop HCID patient transport procedures, including the identification of the emergency medical services (EMS) pick-up location, the internal transport path, including a security protocol that closes off hallways to safely move the patient to the pick-up location, and management of potential waste and terminal cleaning during this process.

Hospital leadership buy-in and support are essential to strengthening HCID preparedness. A considerable amount of staff time and effort, and additional resources are required to develop and plan for an HCID response. As seen in NYC during the 2022 Mpox response, strong C-Suite support involves an active role in these responses, including swiftly coordinating with public health authorities, other healthcare organizations, and communicating key information to hospital staff and the surrounding community [55]. When leadership acknowledges the risks HCIDs pose to the patient, staff, and community, as well as the resources needed to support preparedness and response efforts, it ensures the hospital is well-positioned to respond to the next outbreak. The return on investment of preparedness for an HCID is significant and includes reputational risk reduction, healthcare worker absenteeism and exposure reduction, and community goodwill when safeguards reduce risk to the general population.

The development of successful training programs in NSPS Level 4 facilities will produce an educated and well-informed staff, ultimately reducing potential exposures and thus transmission. Organizations such as NETEC, ASPR TRACIE (Technical Resources, Assistance Center, and Information Exchange), and the RESPTCs have developed and continue to provide additional resources to help support training and education across the NSPS. Online repositories host free tools including sample protocols, training and education strategies, and recorded webinars designed to help hospitals meet the new Joint Commission standards [56,57]. Using these tools, NSPS Level 4 facilities can elevate their preparedness in advance of the next HCID outbreak.

## 5. Conclusions

With the recent establishment of the NSPS, the U.S. is developing crucial infrastructure to robustly respond to emerging and re-emerging pathogens. The COVID-19 pandemic had vast impacts on the U.S., including strain on the healthcare system, changes in how patients interact with the healthcare system, economic instability, and societal changes, all of which the nation continues to recover from years later. The growing threat of H5N1 highlights the vulnerabilities of today’s HCID response system, with all healthcare systems sitting at the frontlines of the response. Minimizing transmission and spread early in an emerging HCID event using the One Health approach can minimize its economic and societal effects. With the new Joint Commission standards for Infection Prevention and Control, along with sustained federal funding for NETEC and the RESPTCs that have developed free HCID resources and training, U.S. healthcare facilities have access to the guidance and tools needed to rebuild their HCID preparedness and response capabilities, stronger than they were in 2019. The next step to ensure national health security is clear: strengthen the readiness and preparedness of our NSPS Level 4 healthcare facilities to mitigate future costly outbreaks. Failing to adequately prepare these facilities will leave healthcare workers and the communities they serve vulnerable to the next inevitable special pathogen outbreak.

## Figures and Tables

**Table 1:** Summary table of key differences between NSPS Level designations.

NSPS Level	Level 1	Level 2	Level 3	Level 4
Treatment Capabilities	Inpatient space for ≥2 pediatric or adult viral hemorrhagic fever (VHF) HCID patients with critical illness Inpatient space for ≥10 HCID patients with airborne transmissible illness Potential for labor and delivery services	Inpatient space for 1–2 VHF HCID patients with critical illness Inpatient space for ≥4 HCID patients with airborne transmissible illness	Identification and Isolation of suspect cases Clinical evaluation and stabilization of 1 VHF patient in isolation space/negative-pressure room Basic care initiation and monitoring	Procedures for screening at facility entry points for respiratory symptoms, fever, rash, and travel history
Facility Type	Adult, pediatric, and/or neonatal care	Adult and/or pediatric focused	Adult and/or pediatric focused	Any healthcare facility
Duration of Care	Duration of illness	Duration of illness	12–36 h	Minimal until transfer is coordinated
PPE Supply	All necessary PPE on hand for ≥2 HCID patients for ≥7 days Plan for resupply to support 21 days of care	Sufficient, appropriate PPE on hand for ≥1–2 HCID patients for ≥7 days Plan for resupply to support 21 days of care	Sufficient, appropriate PPE on hand for up to 3 suspect HCID cases for 12–36 h before resupply	Develop and implement protocols for appropriate PPE donning and doffing techniques and transmission-based isolation precautions
PPE Training	Quarterly	Twice annually	At least annually	Training frequency determined by facility
Exercises	Quarterly	≥2 testing exercises annually for facility plans	≥1 mystery patient exercise annually	Management of HCID cases is incorporated into emergency operation plan(s)
Clinical Lab Ability	Clinical Lab testing to support critical care Dedicated lab space for HCID specimen	Clinical Lab testing to support critical care	Performs point-of-care, on-site clinical diagnostic testing only	Works closely with public health partners to determine next steps for testing patients with suspect or confirmed HCIDs
Waste Management	Clear process to safely manage and dispose of waste Waste Management Training Staff certified in Category A shipping for the collection and shipment of samples to state and CDC labs		Develop and implement waste management protocol for cleaning and disinfecting patient care spaces, surfaces, and equipment	
Decedent Management	Has finalized and coordinated plans with the relevant state agency to manage the remains of an HCID patient who expires in the facility		Has plans to manage the remains of an HCID patient who expires in the facility, including coordination and outreach for technical assistance with the state agency, public health partners, and Level 1 facility	N/A
Research	Develops and maintains institutional processes for requesting investigational drugs while maintaining the standard of care (i.e., emergency investigational new drugs (eINDs)) Participation in NETEC HCID INDs protocols Defines institutional processes for investigational drug research participation	Delivers care under research protocols for HCID patients Institutional processes defined to request study drugs Encouraged participation in NETEC HCID INDs protocols	N/A	N/A

The National Special Pathogens System (NSPS) categorizes healthcare facilities into four levels. This table outlines the differences between each NSPS Level in their care capabilities for patients with a suspected or confirmed high-consequence infectious disease (HCID). N/A—Not Applicable.

## Data Availability

No new data were created or analyzed in this study. Data sharing is not applicable to this article.

## Abbreviations

The following abbreviations are used in this manuscript:

ASPR	Administration for Strategic Preparedness and Response
CARES	Coronavirus Aid Relief and Economic Security
ED	Emergency Department
EMS	Emergency Medical Services
HCID	High-Consequence Infectious Disease
HHS	Health and Human Services
HPAI	Highly Pathogenic Avian Influenza
III	Identify Isolate and Inform
NETEC	National Emerging Special Pathogens Treatment and Education Center
NHE	National Health Expenditures
NSPS	National Special Pathogens System
PPE	Personal Protective Equipment
RESPTC	Regional Emerging Special Pathogen Treatment Center
RETN	Regional Ebola Treatment Network
TRACIE	Technical Resources, Assistance Center, and Information Exchange
UNMC	University of Nebraska Medical Center
VHF	Viral Hemorrhagic Fever
